# A Parallel Multiscale Filter Bank Convolutional Neural Networks for Motor Imagery EEG Classification

**DOI:** 10.3389/fnins.2019.01275

**Published:** 2019-11-26

**Authors:** Hao Wu, Yi Niu, Fu Li, Yuchen Li, Boxun Fu, Guangming Shi, Minghao Dong

**Affiliations:** ^1^Key Laboratory of Intelligent Perception and Image Understanding of Ministry of Education, School of Artificial Intelligence, Xidian University, Xi’an, China; ^2^Engineering Research Center of Molecular and Neuroimaging, Ministry of Education, School of Life Sciences and Technology, Xidian University, Xi’an, China

**Keywords:** EEG, BCI, motor imagery, deep learning, convolutional neural networks

## Abstract

**Objective:**

Electroencephalogram (EEG) based brain–computer interfaces (BCI) in motor imagery (MI) have developed rapidly in recent years. A reliable feature extraction method is essential because of a low signal-to-noise ratio (SNR) and time-dependent covariates of EEG signals. Because of efficient application in various fields, deep learning has been adopted in EEG signal processing and has obtained competitive results compared with the traditional methods. However, designing and training an end-to-end network to fully extract potential features from EEG signals remains a challenge in MI.

**Approach:**

In this study, we propose a parallel multiscale filter bank convolutional neural network (MSFBCNN) for MI classification. We introduce a layered end-to-end network structure, in which a feature-extraction network is used to extract temporal and spatial features. To enhance the transfer learning ability, we propose a network initialization and fine-tuning strategy to train an individual model for inter-subject classification on small datasets. We compare our MSFBCNN with the state-of-the-art approaches on open datasets.

**Results:**

The proposed method has a higher accuracy than the baselines in intra-subject classification. In addition, the transfer learning experiments indicate that our network can build an individual model and obtain acceptable results in inter-subject classification. The results suggest that the proposed network has superior performance, robustness, and transfer learning ability.

## Introduction

Brain–computer interfaces (BCI) establish a direct pathway between the human brain and a computer via brain signal recording and decoding techniques (Lance et al., 2012). Early BCI systems were mainly used for stroke rehabilitation or to improve quality of life for the disabled patients. BCI have been applied to control the devices such as electric wheelchairs (Galán et al., 2008), text spellers (Guan et al., 2005), and prosthetic artificial limbs (Schwartz et al., 2006). Recently, BCI have been widely applied not only for the disabled, but also for healthy people (Lance et al., 2012; Van Erp et al., 2012; Miranda et al., 2015; Saproo et al., 2016). Such BCI are mainly based on non-invasive systems with electroencephalogram (EEG) features, which may be integrated into wearable devices (Mullen et al., 2013, 2015). Functional magnetic resonance imaging (fMRI) based BCI are mainly used in medical treatment (Dong et al., 2014, 2019; Jin et al., 2018). However, it is difficult for such BCI to achieve real-time interaction. In general, BCI contain five major processing steps (Nicolas-Alonso and Gomez-Gil, 2012; Vernon et al., 2018): data collection, preprocessing (Bashashati et al., 2007), feature extraction (Mcfarland et al., 2006), classification (Lotte et al., 2007), and feedback. Because EEG signals have a low signal-to-noise ratio (SNR) and time-dependent covariates, traditional research relies on expert-level experience and prior domain knowledge to design the paradigms and train the classifiers (Schlögl et al., 2005; Wang et al., 2005; Mcfarland et al., 2006; Lotte et al., 2007; Herman et al., 2008; Hsu and Sun, 2009; Suk and Lee, 2013) that would only apply to certain datasets (Wang et al., 2004). It is difficult to extend such strategy to other experiments and datasets (Krepki et al., 2007; Meng et al., 2016).

As a classic paradigm, motor imagery (MI) has been researched and developed for decades. Its physiological basis is that the body movement can produce mu (8–12 Hz) and beta (16–26 Hz) rhythms with event-related (de-)synchronization (ERS/ERD) in the motor sensory areas of the brain. Some research on MI-based devices (such as wheelchairs, prosthetics, and robots) has medical applications and provides human augmentation technologies. The dominant feature extraction algorithms for MI-EEG classification are the common spatial pattern (CSP) and its variants (Ramoser et al., 2000). The idea of CSP is to find a set of spatial filters that optimally discriminate multiple classes of EEG recordings. Benefiting from manual feature selection, filter-bank CSP (FBCSP) (Keng et al., 2012) algorithm selects optimal spatial filters to extract the features. This method has the advantages of simplicity and accuracy. Other CSP-based approaches also extract potentially valuable components of EEG signals after a certain analysis. Unfortunately, EEG features vary over time and change significantly in different individuals (Guger et al., 2003; Blankertz et al., 2010). For new applications of MI, a demand for robust and more general feature extraction techniques is gradually increasing.

Deep learning has made great achievements in computer vision, natural language processing, and speech recognition (Lecun et al., 2015; Schmidhuber, 2015). Currently, end-to-end DL frameworks unify multiple processing stages into one model and build a direct projection from input to output, having demonstrated excellent performance in various tasks (Sutskever et al., 2014; Chan et al., 2016; Redmon et al., 2016). This trend suggests that certain neural computing units, such as convolutional layers in convolutional neural networks (CNNs), can extract implicit features from the signals to improve the performance. The development of DL has also gained interest in the BCI community. Related research includes investigating DL-based models in EEG feature extraction (Li et al., 2015), epilepsy prediction and monitoring (Antoniades et al., 2016; Thodoroff et al., 2016), classification (Bashivan et al., 2015; Vernon et al., 2018), and auditory music retrieval (Stober et al., 2014). DL-based MI is reviewed in detail in the following subsection. However, the application of DL in EEG-based BCI has two challenges: (1) a low SNR and the time-dependent covariates of the EEG signal complicates the feature extraction; (2) insufficient datasets and individual differences in EEG signals among subjects lead to poor performance of transfer learning.

In this paper, we propose a new end-to-end architecture for MI EEG classification. In our layered network architecture, a parallel multiscale filter bank is designed to fully extract the temporal features. Additionally, square and log non-linear operations enhance the non-linear expression ability of the feature reduction layer. To enhance the transfer learning ability, the network initialization and fine-tuning strategy are proposed to train an individual model for inter-subject classification on small datasets. The classification accuracy of the proposed method in the intra-subject experiment is superior to the current well-known end-to-end networks. Inter-subject experiments prove that our proposed network not only obtains competitive results in transfer learning but also has acceptable performance on small datasets.

The rest of this paper is organized as follows. Related work is briefly introduced in section “Related Work.” Section “Materials and Methods” describes the proposed MSFBCNN network in detail. The experiments and results are presented in section “Experiments and Results.” In section “Discussion,” we conclude the paper.

## Related Work

According to the input styles of the networks, DL-based MI is categorized into two types: the feature input network and the raw signal input network.

In the former input style, the MI is accomplished in two stages. First, EEG signals are transformed into vectors by traditional feature-extraction approaches (such as spectrograms, wavelets, and spatial filtering). Next, these feature vectors are fed into the networks. DL is adopted to train a model and classify the features. Kumar et al. (2017) used multilayer perceptrons (MLPs) to replace the traditional support vector machine classifier. Sakhavi et al. (2015) combined CNN and MLP as a new classifier to deal with multiclass MI-EEG tasks. To improve performance of networks, transfer learning and knowledge distillation were explored in which CNN was used as a specific 2D-input classifier (Sakhavi and Guan, 2017). Huijuan et al. adopted augmented-CSP and CNN to discriminate MI-EEG signals, surpassing FBCSP with a novel feature map selection scheme (Yang et al., 2015). Tabar and Halici (2017) fed time-frequency features generated by short-time Fourier transform into a CNN with stacked autoencoders and obtained a competitive accuracy. Bashivan et al. (2015) transformed the temporal EEG into topology-preserving multispectral images and trained a deep recurrent-convolutional network. Zhu et al. (2019) proposed a separated channel convolutional network to encode the multi-channel data. Then, the encoded features are concatenated and fed into a recognition network to perform the final MI task recognition.

The other input style fed time series EEG signals, i.e., the *C* (channel) × *T* (time point) matrices, into deep neural networks directly. Therefore, it is an end-to-end approach. In this network, the steps of feature extraction and classification are combined in a single end-to-end model, with (or without) only minimum preprocessing. The DL model has to learn both an optimal intermediate representation and a classifier for EEG signals in a supervised manner. Several end-to-end models have been proposed and obtained competitive performance in different tasks. As a light network, EEGNet used a few parameters to achieve considerable performance on various EEG classification tasks. Inspired by FBCSP, Schirrmeister et al. (2017) proposed a shallow CNN and a deeper CNN respectively. Both of them yielded higher accuracies compared with FBCSP. Hauke et al. used a simplified CNN model to validate that a DL model was effective in transfer learning tasks for recordings from 109 subjects (Goldberger et al., 2000) without any preprocessing (Dose et al., 2018).

Both input styles have their advantages and disadvantages. The two-stage approach is interpretable and robust, which is guaranteed by handcrafted feature-extraction algorithms. Thus, it is suitable for small training sets and outperforms the traditional methods.

However, the feature input network lost some potential information after the handcrafted feature extraction, which affected the performance. On the contrary, end-to-end models may learn useful features automatically from raw EEG data and achieve satisfactory results. However, for small training datasets, it is hard for the end-to-end methods to train a satisfactory model. As follows from the literature, designing a feasible end-to-end deep neural architecture for MI-EEG classification remains a challenge.

In this paper, to overcome the problem of insufficient number of training samples and improve the robustness of the network, we will focus on the end-to-end style and propose a layered end-to-end network structure of CNNs for MI-EEG signal classification. It is well known that the insufficient number of training samples is prone to cause the overfitting problem of large networks. A common solution is to reduce the scale of network by dropout, network pruning, etc. These tricks work well for the signals with significant features like images and videos. For these signals, the network maybe confused to learn the most general distinguishable features from a small training set, thus one can sacrifice the network capacity to increase the generality and robustness. However, extracting the cerebral activity features from low SNR EEG signal is very challenging. A crude reduction of network connections may decrease the feature extraction capability of network. Therefore, we propose a layered network structure to accomplish the feature extraction task and feature reduction task separately. For feature extraction layer, we propose a MSFBCNN structure to extract sufficient potential features. For feature reduction layer, we adopt a set of non-linear operators followed by dropout connection strategy. In this way, the network is expected to be simplified without loss of feature extraction capacity which fits the characteristic of EEG signals.

## Materials and Methods

In this section, we first introduce the current datasets of MI. Next, a detailed architecture of the proposed network is described. Finally, a training strategy is presented.

### Datasets Description

Currently, there are three publicly available MI-EEG datasets. The main differences of the datasets are the number of channels, trials, subjects, tasks, and sampling rates.

The first two datasets are the BCI Competition IV datasets 2a and 2b (Keng et al., 2012). Both of them have been preprocessed with a band-pass filter between 0.5 and 100 Hz. 2a is a 25-channel [22 EEG and 3 electro-oculogram (EOG)], 4-class MI (left/right hand, feet, and tongue) EEG dataset recorded from 9 different subjects with 250 Hz sampling rate. In the dataset, 9 train sets and 9 test sets are explicitly separated. In the subset, there are 72 trials in each class. The feedback is not provided. 2b is a 6-channel (3 EEG and 3 EOG), 2-class MI (left/right hand) dataset also recorded from 9 different subjects. For each subject, the MI task is separated into five sessions. Unlike the 2a dataset, the first two sessions in the 2B dataset run without feedback, except for the rest sessions.

The last dataset is a high gamma dataset (HGD) (Schirrmeister et al., 2017). In this dataset, 44-channel EEG signals are recorded from 14 subjects with 500 Hz sampling rate. Except subjects 1 and 5, the train sets from the remaining 12 subjects contain over 800 trials, thereby providing comparable data for further experiments.

### Methods

End-to-end CNN has been widely used in MI classification and acquired satisfactory results. To fully use feature information in end-to-end networks to improve their performance, we proposed a layered network that is a feature-extraction network embedded into an end-to-end network.

For example, for a 3-s EEG signal with 22 electrodes and a 250 Hz sampling rate, the size of an input sample is 22 × 750. In end-to-end networks, CNN models process these rectangular EEG matrices and output their class labels. We propose a multilayer end-to-end network that consists of three parts: a front feature extraction layer, feature reduction layer, and classification layer. The detailed network architecture is described as follows.

#### Feature Extraction Layer

The features of EEG data are in two domains: temporal and spatial. Thus, we use separable 2D convolutions with kernel sizes [*k*, 1] and [1, *c*] (where *k* and *c* are integers) to extract temporal and spatial features, respectively. To fully extract temporal features and spatial features, we design a parallel multiscale filter bank convolutional neural network (MSFBCNN). The length of the filter is set manually, depending on the features and the sampling rate of the signal. The extracted temporal features are combined as an input of the spatial convolution after batch normalization. Next, we use a spatial convolution to extract spatial features and reduce dimensions of the feature map. After the spatial convolution, the dimension of EEG channel is squeezed to 1. Both temporal and spatial convolutions expand a third dimension of the feature maps.

#### Feature Reduction Layer

To enhance the non-linear expression ability of the network, we use square and log non-linear functions to extract features that are related to the band power. The temporal dimension and the third dimension are further reduced by a max-pooling layer. Because these operations are in the middle of the model, we call it a feature reduction layer.

#### Classification Layer

The classifier predicts the result after the previous step. As other CNN-based detection networks, the classification is performed by a fully connected layer (Simonyan and Zisserman, 2014).

**FIGURE 1:**
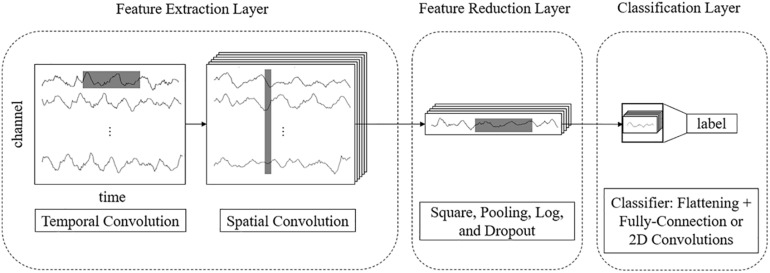
Framework of the proposed MSFBCNN network.

The framework of the proposed MSFBCNN is shown in Figure 1. Unlike other EEG networks [such as EEGNet, ShallowFBCSPNet, and DeepNet (Schirrmeister et al., 2017)] or a simpler CNN model, we select the multiscale temporal convolution to extract the features and design the non-linear function, improving the network expression ability. Furthermore, we can set different learning rates in the layers of three subnetworks to avoid overfitting caused by insufficient data.

**FIGURE 2:**
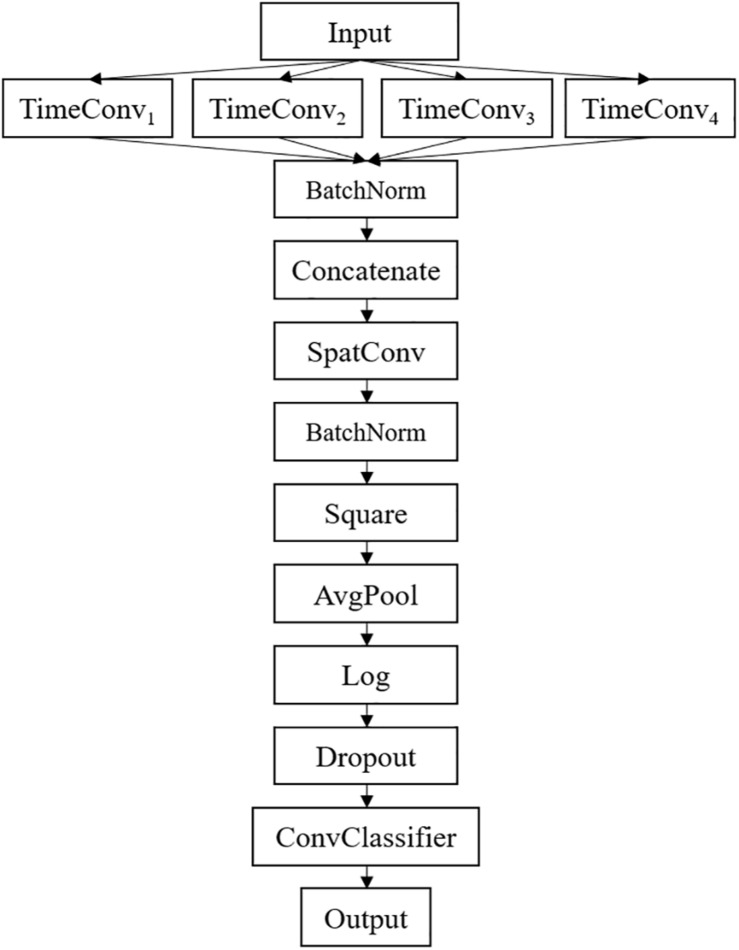
Proposed MSFBCNN architecture.

The detailed network architecture is described in Figure 2 and Table 1. In Table 1, *T* is the number of time points, *C* is the number of channels, *F*_*T*_ is the temporal filter, *F*_*S*_ is the spatial filter, *D* is the ratio of *F*_*T*_ to *F*_*S*_, and *N*_*C*_ is the number of classes. According to the receptive field theory, units in the last module interact with much broader range than that in the temporal convolutional module. Therefore, we simply design multiscale kernels in the temporal convolutional layer inspired by Inception (Szegedy et al., 2016) and Wide-ResNet (Zagoruyko and Komodakis, 2016). This reinforces the capacity of the temporal convolutions of extracting frequency-domain features. We concatenate the output feature maps to feed into the spatial convolutional module. As shown in Table 1, the kernel lengths of temporal filter *F*_*T*_ are set to 64, 40, 26, and 16. The length of the spatial filter is equal to the channel of the EEG data. In the feature reduction layer, the activation of the square and the logarithm operation is non-linear. A dropout layer follows the pooling layer to avoid overfitting, where dropout rate *p* = 0.5. A convolutional classifier outputs the predicted label. For the temporal convolution, the kernel length is likely to be selected arbitrarily.

**TABLE 1:** Detailed architecture of the proposed network.

**Layer**	**# Filters**	**Kernel**	**Stride**	**# Params**	**Output**	**Activation**	**Padding**
Input					(*C*, *T*)		
Reshape					(1, *T*, *C*)		
TimeConv_1_	*F*_*T*_	(64, 1)	(1, 1)	64 * *F*_*T*_	(*F*_*T*_, *T*, *C*)	Linear	Same
TimeConv_2_	*F*_*T*_	(40, 1)	(1, 1)	40 * *F*_*T*_	(*F*_*T*_, *T*, *C*)	Linear	Same
TimeConv_3_	*F*_*T*_	(26, 1)	(1, 1)	26 * *F*_*T*_	(*F*_*T*_, *T*, *C*)	Linear	Same
TimeConv_4_	*F*_*T*_	(16, 1)	(1, 1)	16 * *F*_*T*_	(*F*_*T*_, *T*, *C*)	Linear	Same
Concat					(4 * *F*_*T*_, *T*, *C*)		
BatchNorm				2 * *F*_*T*_	(4 * *F*_*T*_, *T*, *C*)		
SpatialConv	*F*_*S*_	(1, *C*)	(1, 1)	C * 4 * *F*_*T*_ * *F*_*S*_	(*F*_*S*_, *T*, 1)	Linear	Valid
BatchNorm				2 * *F*_*S*_	(*F*_*S*_, *T*, 1)		
Non-Linear					(*F*_*S*_, *T*, 1)	Square	
AveragePool		(75, 1)	(15, 1)		(*F*_*S*_, *T*//15, 1)		Valid
Non-Linear						Log	
Dropout					(*F*_*S*_, *T*//15, 1)		
Classifier	*N*_*C*_	(*T*//15, 1)	(1, 1)	*F*_*S*_ * (*T*//15) * *N*_*C*_	*N*_*C*_	Linear	Valid

### Network Training

In the proposed MSFBCNN, we use a network training algorithm similar to those of CNNs. As for MI classification, the categorical cross-entropy loss function is defined as:

(1) $$C\,\left(p,q\right)=-\sum_{i}p_{i}\,\log q_{i},i=1,2,\cdots ,n$$

where *p* is the target distribution, *q* is the observed distribution, and *n* is the number of classes. We use the Adam method (Kingma and Ba, 2014) for optimization.

MI datasets have two drawbacks: (1) the number of samples is insufficient; (2) the features of the EEG data are different for each subject. The current MI networks are designed, trained, and tested for specific datasets. To improve the robustness of our proposed network, we design two strategies to enhance the transfer learning ability.

#### Network Initialization

The convolution layer weights are initialized using the normal distribution with zero mean and unit variance. The batch normalization layer weights use 1 for initialization. The learning rate is 1e−3, and the decay weight is 1e−7. The batch size is 64. These initialization parameters are acquired from the experiment in advance.

#### Fine-Tuning

First, we use the initial parameters to train the proposed network and acquire a coarse model from the open dataset. Next, for a specific subject dataset, the individual training data are mixed with a randomly selected open dataset and a validation set for further training. To avoid overfitting, we use a layered learning rate in the calibration experiment. The learning rates of the feature extraction layer, feature reduction layer, and classification layer are 1/27, 1/9, and 1/3 of the default learning rate, respectively.

Obviously, the coarse model has learned a general classifier. However, the fine-tuning strategy can further help the network to match the specific pattern of the individual subject. This strategy expands our training data and improves the transfer ability. Subsequently, the classifier is fine-tuned to match the specific pattern that relies on data from a specific subject.

## Experiments and Results

To verify the feasibility and performance of our proposed method, we conduct a series of experiments for MI classification. These experiments are run upon the Braindecode framework, which is supported by PyTorch.

The three datasets described in section “Datasets Description” are used for classification. Because the data collecting paradigms of the three datasets are similar, we extract the data of an epoch between 0 and 4.5 s after the corresponding trial starts from all datasets. To keep the same sampling rate for the first two datasets, the EEG recordings in HGD are resampled to 250 Hz. We do basic preprocessing, such as frequency filtering and normalization, to augment the SNR of the EEG data. All datasets are denoised by a low-pass filter of 38 Hz and a high-pass filter of 4 Hz. The amplitude of all EEG recordings is normalized by an exponential weighted moving average.

### Intra-Subject Classification

The intra-subject classification experiment is a general benchmark to verify the performance of the proposed network for an individual subject. Each EEG dataset is divided into a train set, validation set, and test set. We use three state-of-the-art networks as baselines: DeepNet, EEGNet, and ShallowFBCSPNet. The same pipeline is used for all methods. The average accuracy of each network is collected after each model is trained and tested 10 times. The results are shown in Table 2. Table 2 shows that our proposed MSFBCNN network acquires the best results on all datasets compared with the baselines. This is because our proposed network can fully extract the temporal features thanks to multiscale filter banks and the outstanding non-linear expression ability.

**TABLE 2:** Accuracy in intra-subject experiments.

	**Dataset**		
	
**Network**	**2a**	**2b**	**HGD**
DeepNet	66.8	83.6	84.8
EEGNet	66.7	83.1	84.0
ShallowFBCSPNet	72.3	81.5	91.6
MSFBCNN	75.8	84.3	94.4

### Inter-Subject Transfer Learning

To verify the transfer ability of the proposed method, we have conducted the inter-subject transfer experiment. In this experiment, EEG recordings from other subjects are used to train a model in advance. Next, the fine-tuning strategy is adopted to further train the individual model. To verify the generality of the proposed fine-tuning strategy, we use this strategy for DeepNet, EEGNet, and ShallowFBCSPNet on three datasets. The results are shown in Table 3. Our proposed method has a higher accuracy than the baselines in inter-subject transfer learning. In addition, compared with the intra-subject experiment, the performance of all networks is improved after the fine-tuning, which proves that the proposed strategy is effective.

**TABLE 3:** Accuracy of inter-subject transfer learning.

	**Dataset**		
	
**Network**	**2a**	**2b**	**HGD**
DeepNet	71.9	84.1	90.9
EEGNet	69.9	83.6	88.6
ShallowFBCSPNet	73.8	83.7	92.3
MSFBCNN	75.9	84.7	94.9

### Transfer Learning on Small Datasets

Classification remains a challenge for a small number of training samples, because it is difficult for a model to learn the full distribution from insufficient data. This experiment verifies the performance of the proposed method for transfer learning on small datasets. It is a desideratum to use as few training samples as possible to achieve a satisfactory accuracy of the classification model. In this experiment, we select 10, 20, 50, and 100 samples, which are used in the inter-subject transfer learning experiment for fine-tuning. The classification accuracy results are shown in Table 4. The accuracy increases with the increase of the sample size. Moreover, we also find that our method obtains acceptable results with 100 samples compared with the inter-subject experiment in 2a and 2b datasets, which proves that the proposed fine-tuning strategy can enhance the transfer learning ability on small datasets.

**TABLE 4:** Results of fine-tuning on a small number of training samples.

	**Dataset**		
	
**Training samples**	**2a**	**2b**	**HGD**
10	60.0	74.8	84.7
20	65.6	78.9	84.9
50	67.6	81.8	86.8
100	75.0	83.1	89.3

### Effects of Parallel Multiscale Filter Bank in Different Models

Existing MI networks are designed, trained and tested for specific datasets. As shown in Table 2, compared with DeepNet and EEGNet, ShallowFBCSPNet has better performance on 2a and HGD dataset but not on 2b dataset. In the proposed method, we introduce a parallel MSFBCNN for EEG-based BCI. The results on all of the three datasets prove that the performance of our MSFBCNN is better than other MI networks, which verifies that our method is robust on datasets.

In the proposed method, we design a parallel multiscale filter bank convolution in our network. The essential is to fully extract potential features. To verify the validity of the parallel multiscale filter bank convolution structure, we embed the structure in EEGNet and DeepNet, named as T-EEGNet and T-DeepNet, respectively. We also carry out the experiments on the three datasets. The results are shown in Table 5. According to Table 5, impressive improvements are acquired compared with the original EEGNet and DeepNet after the adoption of the parallel multiscale filter bank convolution.

**TABLE 5:** Comparison of EEGNet, DeepNet, T-EEGNet, and T-DeepNet.

	**Dataset**		
	
**Network**	**2a**	**2b**	**HGD**
EEGNet	66.7	83.1	84.0
T-EEGNet	70.8	85.3	91.7
DeepNet	68.8	83.6	88.1
T-DeepNet	70.9	84.9	92.8

Although ShallowFBCSPNet has a parallel filter bank convolution, the kernel size is constant. It ignores the effect of multiscale on feature extraction. As shown in Table 2, our proposed method performs better than ShallowFBCSPNet.

## Discussion

Our work is devoted to designing and training an end-to-end network to fully extract temporal and spatial features from EEG signals. Compared with other three networks, we add a parallel multiscale filter bank convolution to our network and acquire impressive improvements. In addition, we embed the parallel multiscale filter bank convolution structure in EEGNet and DeepNet to verify the validity of the proposed structure.

In order to explore the parameter impact on the results, we did some experiments. There are two hyper-parameters in the proposed technique, *F*_*T*_ and *F*_*s*_ which denote the kernel number of temporal convolution and spatial convolution, respectively. To optimize the network, we have to search all the possible combinations of *F*_*T*_ and *F*_*s*_. To accelerate the greedy search process, we introduce an intermediate parameter *D* which is the merchant of *F*_*T*_ and *F*_*s*_. Like EEGNet, we enumerate a small range of *D* firstly, and adjust *F*_*s*_ with fixed *D*. For a comprehensive comparison work, we traverse the super-parameters of EEGNet. Effects of different *F*_*T*_ and *D* with comparison to the original are shown in Table 6. It can be observed that, the proposed MSFBCNN technique outperforms EEGNet’s best performances in most of the cases. With the setup *F_*T*_* = 40, *D* = 1 MSFBCNN achieves the best performances (75.8%).

**TABLE 6:** Effects of different *F*_*T*_ and *D* with comparison to the original.

			**2a**	**2b**
MSFBCNN	*F*_*T*_	10	64.5	81.0
		20	67.9	80.8
		50	67.2	80.0
		80	66.6	79.9
	*D*	0.5	64.4	80.5
		2	65.3	80.0
		*F*_*T*_ = 40, *D* = 1	75.8	84.3
EEGNet	*F*_*T*_	4	61.5	79.2
		16	63.6	77.1
		32	58.4	75.0
		40	56.2	72.0
	*D*	1	63.5	78.1
		4	55.4	76.8
		*F*_*T*_ = 8, *D* = 2	66.7	83.1

**FIGURE 3:**
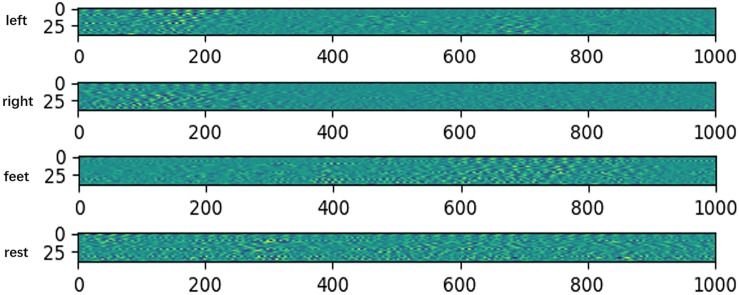
Features map after extracting temporal feature on HGD. The *x*-axis denotes time, and the *y*-axis denotes the channel.

To verify the network extracted features are valid, we do some feature visualizations. We plot the features map after extracting temporal feature on HGD, which is a 4-class MI (left/right hand, feet, and rest) dataset. The results are shown in Figure 3.

The visualization results show that the feature distributions are different from each other among different tasks. The extracted features of left-hand and right-hand tasks are mainly distributed in the 200 ms after the imaging, but they are different in channels. The feature of left-hand imagination is distributed from channel 0 to channel 25, while the feature of left-hand is mainly distributed in the channel from 25 to 44. The feature of feet task arises in the rear part of the 1 s dataset. The feature of rest task is distributed in all of the 1 s dataset.

Benefitting from the fine-tuning strategy with multilayer end-to-end structure, we can easily set layered learning rate for each of the three parts to avoid overfitting caused by insufficient data. In section “Inter-Subject Transfer Learning,” we conduct the inter-subject transfer learning experiment. Compared with the intra-subject experiment results in Table 2, the performances of all networks in Table 3 are improved after the fine-tuning, which proves that the proposed strategy is effective.

In transfer learning on small dataset experiment, our method obtains acceptable results with 100 samples compared with the inter-subject experiment in 2a and 2b datasets, which proves that the proposed fine-tuning strategy can enhance the transfer learning ability on small datasets.

Training network consumes a rather long time, but the network initialization and transfer learning on small dataset strategy can help us to build an individual model quickly on small training samples. After the training, the proposed method only takes 0.0128 s for prediction. Thus, we can build an online MI system to control the devices such as electric wheelchairs and prosthetic artificial limbs. Furthermore, we can use the proposed method on MI driver assistant system and human-machine collaborative system to improve the abilities of human.

The current work is mainly evaluated on datasets but not online. In our future work, we will further improve the efficiency transfer learning on small dataset experiment and try to build online human-machine collaborative system.

## Conclusion

In this study, we propose a parallel MSFBCNN for EEG-based BCI, which can fully extract potential features from EEG and obtain an outstanding model in the presence of limited data. We introduce our layered end-to-end network structure in detail. The proposed structure has three parts: the front feature extraction layer, feature reduction layer, and classification layer. To enhance the transfer learning ability, we propose a network initialization and fine-tuning strategy for training the network. Finally, we compare our MSFBCNN with the state-of-the-art approaches for intra-subject classification. The classification accuracy indicates that our method outperforms the baselines. Additionally, inter-subject and small dataset experiments verify that our fine-tuning strategy can meet the transfer learning demands and obtain acceptable results.

## Data Availability Statement

The datasets 2a and 2b for this study can be found in the http://www.bbci.de/competition/iv/. The datasets HGD can be found in the https://github.com/robintibor/high-gamma-dataset/.

## Author Contributions

HW processed and analyzed the data, and wrote the manuscript. HW and YL developed the parallel multiscale filter bank convolutional neural network. BF helped in data analysis. YL helped in manuscript editing. FL, YN, and MD supervised the development of work, helped in manuscript editing and evaluation. FL and GS designed the research content and research direction.

## Conflict of Interest

The authors declare that the research was conducted in the absence of any commercial or financial relationships that could be construed as a potential conflict of interest.
